# Treatment of COVID-19 patients with quercetin: a prospective, single center, randomized, controlled trial

**DOI:** 10.3906/biy-2104-16

**Published:** 2021-08-30

**Authors:** Hasan ÖNAL, Bengü ARSLAN, Nurcan ÜÇÜNCÜ ERGUN, Şeyma TOPUZ, Seda YILMAZ SEMERCİ, Mehmet Eren KURNAZ, Yulet Miray MOLU, Mehmet Abdussamet BOZKURT, Nurettin SÜNER, Ali KOCATAŞ

**Affiliations:** 1 Department of Pediatric Nutrition and Metabolism Clinics, İstanbul Kanuni Sultan Süleyman Training and Research Hospital, İstanbul Turkey; 2 Department of Neonatology, İstanbul Kanuni Sultan Süleyman Training and Research Hospital, İstanbul Turkey; 3 Department of General Surgery, İstanbul Kanuni Sultan Süleyman Training and Research Hospital, İstanbul Turkey; 4 Department of General Medicine, İstanbul Kanuni Sultan Süleyman Training and Research Hospital, İstanbul Turkey

**Keywords:** Bromelain, Covid-19, quercetin, vitamin C

## Abstract

Scientific research continues on new preventive and therapeutic strategies against severe acute respiratory syndrome Coronavirus-2 (SARS-CoV-2). So far, there is no proven curative treatment, and a valid alternative therapeutic approach needs to be developed. This study is designed to evaluate the effect of quercetin in COVID-19 treatment. This was a single-centre, prospective randomized controlled cohort study. Routine care versus QCB (quercetin, vitamin C, bromelain) supplementation was compared between 429 patients with at least one chronic disease and moderate-to-severe respiratory symptoms. Demographic features, signs, laboratory results and drug administration data of patients were recorded. The endpoint was that QCB supplementation was continued throughout the follow-up period from study baseline to discharge, intubation, or death. The most common complaints at the time of hospital admission were fatigue (62.4%), cough (61.1%), anorexia (57%), thirst (53.7%), respiratory distress (51%) and chills (48.3%). The decrease in CRP and ferritin levels was higher in the QCB group (all Ps were < 0.05). In the QCB group, the increase in platelet and lymphocyte counts was higher (all Ps were < 0.05). QCB did not reduce the risk of events during follow-up. Adjustments for statistically significant parameters, including the lung stage, use of favipiravir and presence of comorbidity did not change the results. While there was no difference between the groups in terms of event frequency, the QCB group had more advanced pulmonary findings. QCB supplement is shown to have a positive effect on laboratory recovery. While there was no difference between the groups in terms of event frequency, QCB supplement group had more advanced pulmonar findings, and QCB supplement is shown to have a positive effect on laboratory recovery/results. Therefore, we conclude that further studies involving different doses and plasma level measurements are required to reveal the dose/response relationship and bioavailability of QCB for a better understanding of the role of QCB in the treatment of SARS CoV-2.

## 1. Introduction

Scientific research continues on new preventive and therapeutic strategies against severe acute respiratory syndrome Coronavirus-2 (SARS-CoV-2). So far, there is no proven curative treatment for the novel coronavirus disease 2019 (COVID-19), and while vaccination is continuing worldwide, “wild” protocols based on “ancient” anti-inflammatory and anti-viral drugs are being offered. A valid and alternative therapeutic approach needs to be developed.

Affecting the nasopharyngeal cells first, SARS-CoV-2 can target different tissues such as lung, vascular endothelium, kidney and nervous system at various degrees, and it can cause severe illness and death (Russo et al., 2017; Spagnuolo et al., 2018). With the advantage of the lack of systemic toxicity, flavonoids, including quercetin are proven to potentialize the effects of routine drugs against coronavirus (Ferrer et al., 2008). Flavonoids owe their antioxidant, anti-inflammatory and anti-viral properties against a wide range of DNA and RNA viruses, to their pleiotropic molecular structure that acts by targeting variable cells on multiple pathways (Puelles et al., 2020). Quercetin, as a widely available plant flavonoid, has antioxidant, anti-inflammatory, antiviral and immunoprotective effects (Nair et al., 2002; Robaszkiewicz et al., 2007; Uchide et al., 2011). It is thought as one of the molecules that is responsible for the cardiovascular protective effect of Mediterranean diet (Gormaz et al., 2015). A wealth of literature supporting the anti-viral properties of quercetin in both in vitro and in vivo experiments exists (Ishitsuka et al., 1982; Kaul et al., 1985; Evers et al., 2005; De Palma et al., 2008; Zandi et al., 2011; Ranucci et al., 2020).

Severe acute respiratory syndrome coronavirus (SARS-Cov) was first identified in 2003. Protease activity plays a role in the attachment and replication of this virus to the cell surface. Quercetin has been shown to inhibit its proteolytic activity by binding to virus-specific protease (Chen et al., 2006). The SARS-CoV-2 receptor binding site is similar to the binding site of SARS-CoV, as well as the SARS-Cov-2 protease, that was defined as the binding site for the hydroxyl groups of quercetin and its derivatives (Rota et al., 2003; Zhang et al., 2020). Besides, viral S-protein of SARS-CoV-2 infects the human cell via binding angiotensin-converting enzyme-2 (ACE-2) receptor. This mechanism of the virus emerged as a target for several anti-viral therapies. A supercomputer modeling study using the world’s most powerful supercomputer, SUMMIT, identified several candidate small molecule drugs, which bind to either the isolated SARS-CoV-2 Viral S-protein at its host receptor region or to the S protein-human ACE2 interface (Smith et al., 2020). Interestingly, in this study, quercetin was identified among the top five scoring ligands for viral S-protein-human ACE2 receptor interface. Hence, quercetin is thought to be a good candidate to enhance the impact of routine treatment of COVID-19. Since pure quercetin has a poor bioavailability, a combination with vitamin C and bromelain can help to solve this issue. Therefore, the aim of this study is to determine whether a combination of quercetin with vitamin C, and bromelain had a curative role in the treatment of COVID-19.

## 2. Material and methods

### 2.1. Design

This was a single-centre, prospective, randomized controlled cohort study. This study was conducted in Health Sciences University Kanuni Sultan Suleyman Training and Research Hospital, which was designated as a pandemic hospital. The Ministry of Health and local ethics committee approved the study (Ethics Committee approval number: KAEK/2020.05.50). 

### 2.2. Participants 

Between May 7 and July 8, 2020, adults who were hospitalized in the pandemic ward with the diagnosis of COVID-19 were included upon written individual informed consent. All participants were evaluated with nasopharyngeal swab polymerase chain reaction (PCR) and chest computed tomography (CCT). The standart treatment protocol recommended by the Ministry of Health was applied for all cases. The recommended treatment regimen is hydroxychloroquine, 400 mg daily for another 5 days, and favipiravir, 2 × 600 mg for 4 days following a 2 × 1600 mg loading dose on day one. QCB (1000mg quercetin, 1000 mg vitamin C and 100 mg bromelain) supplementation was added daily in 2 divided doses to 52/447 patients with at least one chronic disease and moderate-to-severe respiratory symptoms. Computer-generated random numbers were used for simple randomization. Exclusion criteria determined as severe respiratory failure, shock and combined failure of other organs that required ICU (intensive care unit) monitoring and treatment, previous history of allergic reactions against any component of QCB, pregnant or lactating women, women of childbearing age with a positive pregnancy test, breastfeeding, miscarriage, or within 2 weeks after delivery, and participation in another clinical trial against SARS-CoV-2 treatment currently or in the past 28 days.

The study was reported according to the Consolidated Standards of Reporting Trials guidelines and registered on ClinicalTrials.gov (number: NCT04377789) on March 20, 2020. The primary endpoint of the study was determined as QCB supplementation was continued throughout the follow-up period from study baseline to discharge, intubation, or death. Demographic features, vital signs, laboratory test results during follow-up, drug administration data, past, and current diagnoses of the patients were recorded. CCT findings of the cases were evaluated in 5 stages: stage 0 is the lung being completely normal; stage 1, light one-sided ground glass image; stage 2, multifocal double-sided ground glass image; stage 3, multifocal bilateral ground glass and stage 4, opacity, air bronchogram, bilateral ground glass and opacity, respectively.

## 3. Statistical analysis

The quantitative data were described as the mean ± standard deviation (SD) in case of normal distribution, or as the median (min-max) otherwise. A sample size calculation was performed based on our observed results by using a one-sided McNemar’s test. A sample size of 429 individuals, at least 49 in each arm, is found to be sufficient to detect a clinically significant difference between groups with 80% power and a 5% level of significance. The qualitative data were described by the number of cases (proportion, %). Patient characteristics were compared using the χ2 test or Fisher›s exact test for categorical data and the Mann–Whitney U test for continuous data. Cox proportional-hazards regression models were used to estimate the association between QCB use and the composite endpoint of intubation or death. Statistical significance was set when the probability (P) value was < 0.05 and changes were referred to as significant at this P-value.

## 4. Results

**Figure 1:**
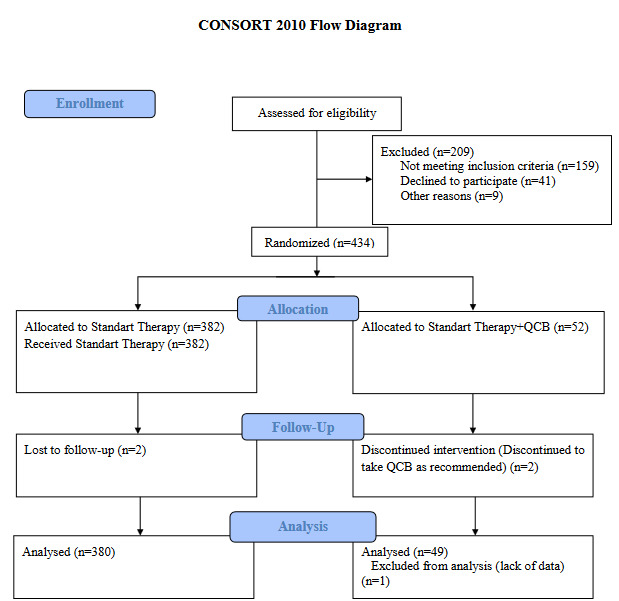
Flow diagram of the study.

A total of 429 adult covid patients hospitalized in the COVID ward were included in the study between May 7 and July 8, 2020. Flow chart of the study was demonstrated as Figure-1 (Figure 1). None of the adverse effects related to QCB supplement was observed in participants.

The most common symptoms at the time of hospital admission were fatigue (62.5%), cough (60.4%), anorexia (56.6%), thirst (54.3%), respiratory distress (51%) and chills (48.3%; Table 1).

**Table 1:** Symptoms of all patients at the time of admission.

Symptoms	N = 429 (%)
Fatigue	268 (62.5)
Cough	259 (60.4)
Poor appetite	243 (56.6)
Thirst	233 (54.3)
Respiratory distress	219 (51.0)
Chill	207 (48.3)
Headache	203 (47.3)
Joint pain	165 (38.5)
Nausea	158 (36.8)
Insomnia	156 (36.4)
Back pain	154 (35.9)
Fever	152 (35.4)
Sore throat	129 (30.1)
Vertigo	127 (29.6)
Diarrhae	126 (29.4)
Chest pain	125 (29.1)
Loss of taste	117 (27.3)
Vomiting	98 (22.8)
Muscle pain	96 (22.4)
Loss of smell	87 (20.3)
Syncope	13 (3.0)

Lymphopenia was detected in 20,7%, thrombocytopenia in 4.2%, elevated values of CRP in 32.6%, LDH in 54.3%, D-Dimer in 26.3% and ferritin in 31.3% of the patients included in the study (Table 2).

**Table 2:** Labratory parameters of all patients at the time of admission.

	N = 429 (%)
LDH (>250 U/L)	233 (54.3)
CRP (>40 mg/dL)	140 (32.6)
Ferritin (>300ng/mL)	134 (31.3)
D-Dimer (>1mg/dL)	113 (26.3)
Lymphopenia (<100/mm^3^)	89 (20.7)
Thrombocytopenia (<120k/mm^3^)	18 (4.2)

There was no significant difference between the standard treatment group and the standard treatment plus QCB group in terms of age and sex (p = 0.22; p = 0.16) (Table 3). In terms of comorbid diseases, the standard treatment plus QCB group had a significantly higher number of chronic obstructive pulmonary disease (COPD, p = 0.02), though there was no significant difference in terms of other diseases. Both groups did not differ in terms of smoking (p = 0.43; Table 3). Pulmonary findings at the time of hospital admission in the standard therapy plus QCB group were significantly more severe than in the standard therapy group (p = 0.03; Table 3). The proportion of patients with an oxygen saturation <93 mmHg at admission and/or follow-up was significantly higher in the group receiving standard therapy plus QCB (p = 0.016; Table 3). Nasopharyngeal swab SARS CoV2 PCR result was positive in 40%–50% of cases for both groups (p = 0.84; Table 3).

**Table 3:** Comparison of demographic characteristics of the groups.

		Standard treatment Group n (%)	Standard treatment plus QCB Group n (%)	
N		380	49	P
Standard therapy				
Hydroxychloroquine		372 (97.9)	46 (93.9)	0.12
Favipiravir		40 (10.5)	14 (28.6)	0.001
Sex				
	Male	210 (55.3)	32 (65.3)	
	Female	170 (44.7)	17 (34.7)	
Age				0.16
	18-30	20 (5.3)	0 (0)	
	30-40	38 (10)	1 (2)	
	40-50	78 (20.5)	9 (18.4)	
	50-60	92 (24.2)	16 (32.7)	
	60-70	78 (20.5)	13 (26.5)	
	70-80	39 (10.3)	8 (16.3)	
	80-90	29 (7.6)	2 (4.1)	
	90-100	6 (1.6)	0 (0)	
Comorbidities				
	COPD	20 (5.1)	7 (13.5)	0.02
	Asthma	50 (13.2)	10 (19.2)	0.19
	Cardiac disease	82 (21.6)	15 (30.6)	0.20
	Hypertension	146 (38.4)	23 (46.9)	0.27
	Diabetes Mellitus	110 (28.2)	16 (32.7)	0.50
	Malignity	12 (3.2)	2 (4.1)	0.66
	Obesity	3 (0.8)	1 (2)	0.38
	Rheumathologic Disease	20 (5.3)	3 (6.1)	0.73
	Chronic liver disease	2 (0.5)	0 (0)	1
	Chronic renal disease	0 (0)	1 (2)	0.11
Smoking				0.43
	-	220(57.9)	25 (51)	
	+	34 (8.9)	7 (14.3)	
	Past history of smoking	126 (33.2)	17 (34.7)	
CCT at admission				0.03
0: Totally normal 1: Slight, one-sided ground-glass 2-Multifocal two-sided ground-glass 3- Multifocal two-sided ground-glass and opasity 4- Air bronchogram, bilateral ground-glass and opacity	0	25 (6.6)	2 (4.1)	
	1	46 (12.2)	0 (0)	
	2	121 (32)	13 (26.5)	
	3	156 (41.3)	28 (57.1)	
	4	30 (7.9)	6 (12.2)	
Partially Oxygen saturation				0.016
	>93	263 (66.6)	26 (50)	
	<93	132 (33.4	26 (50)	
				
SARS-CoV-2 test result				0.84
	Positive	175 (46.1)	21 (42.9)	
	Negative	189 (49.7)	25 (51.0)	
	Test result not yet known	16 (4.2)	3 (6.1)	

**Figure 2:**
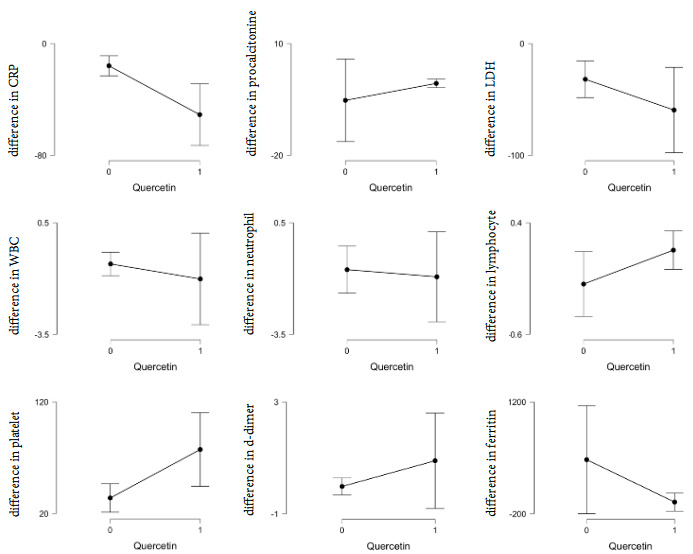
Alterations in acute phase reactants and complete blood count parameters during the follow-up.

The decrease in the levels of C-reactive protein and ferritin was significantly higher in the group that received standard treatment plus QCB compared to the other group (Pcrp = 0.001; Pferritin = 0.033; Table 4, Figure 2). Also, the increase in thrombocyte and lymphocyte count was significantly higher in the group receiving standard therapy plus QCB (Pplatelet = 0.006, Plymphocyte = 0.014; Table 4). It was found that the addition of QCB to the standard therapy/routine care did not reduce the risk of events during the service follow-up period (Omnibus tests of model coefficients p = 0.04, Hazard Ratio: 0.19, p = 0.11, (0.02–1.48); Table 5).

**Table 4:** Comparison of groups in terms of laboratory parameters.

		Standard treatment	Standard treatment plus QCB	P
		Median (min-max)	Median (min-max)	
C-reactive protein (mg/L)				
	1.measurement	21.7 (0.38–353)	49.18 (4.6–339)	
	2.measurement	13.3 (0.30–326)	22.23 (0.4–88.8)	
	Difference between the results (2-1)	–2.10	–34.6	0.001
Procalsitonin (ng/mL)				
	1.measurement	0.08 (0.02–305)	0.13 (0.05–10.5)	
	2.measurement	0.06 (0.00–11)	0.06 (0.02–1.78)	
	Difference between the results (2-1)	0.00	–0.06	0.28
LDH (U/L)				
	1.measurement	271 (20–1247)	313 (140–556)	
	2.measurement	241 (41–955)	230 (37–566)	
	Difference between the results (2-1)	–33	–55	0.21
Hgb				
	1.measurement	13.4 (5.5–17.5)	13.3 (8.9–16)	
	2.measurement	12.5 (4.2–20.5)	12.8(8.5–16)	
	Difference between the results (2-1)	–0.7	–0.8	0.45
Leukocyte count per mm^3^				
	1.measurement	6.6 (0.4–39.3)	7.36 (3.1–43.0)	
	2.measurement	5.9 (2.1–21.6)	7.58 (4.0–18.9)	
	Difference between the results (2-1)	–0.68	–0.5	0.47
Neutrophil count per mm^3^				
	1.measurement	4.21 (0.01–48.4)	4.73 (0.9–40.0)	
	2.measurement	3.57 (1.0–40.8)	4.8 (2.3–17.9)	
	Difference between the results (2-1)	–0.51	–0.08	0.42
Lymphocyte count per mm^3^				
	1.measurement	1.50 (0.00–26.8)	1.30 (0.5–3.80)	
	2.measurement	1.50 (0.3–21.5)	1.60 (0.4–3.5)	
	Difference between the results (2-1)	–0.10	0.10	0.010
Platelet count per mm^3^				
	1.measurement	214 (9–768)	242 (114–471)	
	2.measurement	243 (4–698)	315 (64–687)	
	Difference between the results (2-1)	15.5	69	0.006
D-dimer (mg/dL)				
	1.measurement	0.72 (0.2–24.8)	0.88 (0.19–7.1)	
	2.measurement	0.71 (0.17–14.0)	0.79 (0.17–35.2)	
	Difference between the results (2-1)	–0.03	0.08	0.14
Ferritin (ng/mL)				
	1.measurement	217 (3.9–1514)	362 (65.9–2166)	
	2.measurement	268 (3.3–1986)	384 (57–1621)	
	Difference between the results (2-1)	22.4	–8.1	0.033

**Table 5:** Average time of hospital follow-up, discharge rate and rate of events of the groups.

		Standard treatment	Standard treatment and QCB
		Median (min-max)	Median (min-max)
Outcomes			
Follow-up (days)		6 (2–57)	8 (2–30)
Discharge		360 (94.7)	48 (98.0)
Events			
	Need for intensive care	14 (3.7%)	0 (0)
	Respiratory failure (n = 8)		
	Stroke (n = 3)		
	Acute Myocard Infarct (n = 2)		
	Delirium, encephalytis (n = 1)		
	Death in the ward	6 (1.6%)	1 (2%)(Acute myocard infarct)

After adjustment for the conditions (CCT lung involvement stage, oxygen saturation, favipiravir use, presence of comorbid chronic disease), similar results were observed between the groups (statistically significantly different values were persisted same as the previous).

## 5. Discussion

This study is unique of revealing the potential effect of QCB on the treatment of COVID-19. Quercetin, as a common flavonoid of many fruits and vegetables such as high capers, lovage, and tea (Camellia sinensis), is proven to inhibit SARS-CoV-2 binding to the human cell via virus-specific protease and viral S-protein S-human ACE-2 interface (Nair et al., 2002; Uchide et al., 2011; Gormaz et al., 2015).

In a study of Al Shukor and colleagues, quercetin and epicatechin were able to form an interaction with ACE via both the zinc ion of ACE together with amino acids of ACE. The study also showed that the presence of a catechol group on the flavonoid seemed to increase the potency to inhibit ACE (Al Shukor et al., 2013). Therefore, quercetin seemed to have the most ACE inhibiting capacity between all flavonoids.

Recent publications from different centers show that COVID19 infection goes with endothelitis and increased hypercoagulation. It affects many organ systems in the body, including the lung (Ackermann et al., 2020; Bowles et al., 2020; Bösmüller et al., 2020; Hanley et al., 2020; McFadyen et al., 2020; Potus et al., 2020; Zhang et al., 2020).

In the literature, platelet activation and aggregation have been reported in patients with severe COVID-19, but the triggers of these processes are not discussed yet (Hottz et al., 2020; Salamanna et al., 2020). Most recently, platelets were reported to trigger degranulation of perivascular mast cells leading to inflammatory responses and tissue injury (Karhausen et al., 2020). Moreover, mast cell degranulation associated with interstitial edemaand immunothrombosis was just reported in alveolar septa of deceased patients with COVID-19 (Motta Junior et al., 2020). Quercetin is one of the potential anti-COVID-19 drugs with its inhibitory effect on platelet aggregation and mast cell activation (Ross JA and Kasum CM, 2002). Isoquersetin, a derivative of quercetin with a 5-fold increased intestinal absorption, has been shown in a phase II clinical study to significantly reduce D-Dimer levels by inhibiting disulfide isomerase (PDI) and preventing blood clotting in metastatic late-stage cancer patients (Zwicker et al., 2019). PDI is one of the factors that actives the coagulation factors secreted by thrombocyte and endothelial cells in case of vascular injury.

US Food and Drug Administration described quercetin as GRAS status (generally recognized as safe) (Davis et al., 2009). In this study, a combination of quercetin, vitamin C and bromelain (QCB) was given to our patients, instead of pure quercetin. The main reason for this is that the bioavailability of quercetin is highly variable, ranging from 0% to 50% (Graefe EU al., 1999). When oral quercetin is taken 500 mg, the maximum plasma concentration is reached within 4 h (Moon et al., 2008). Oral supplementation with quercetin up to 1 g/day for 3 months has not resulted in significant adverse effects (Harwood et al., 2007). In case of glutathione and ascorbate deficiency, quercetin can have a prooxidant effect (Boots et al., 2007). For this reason, a combination of quercetin and vitamin C is recommended in severe infections where the body is under stress (Kim et al., 2013). Vitamin C exhibits immunomodulatory activity, increases interferon production through STAT3 phosphorylation, limits organ damage caused by cytokines, supports survival in fatal infections, and most importantly, it can recycle oxidized quercetin (Li et al., 2006; Askari et al., 2012; Kim et al., 2013; Valero et al., 2015). SARS-Cov-2 infection initiates a strong inflammatory and erratic reaction with the “cytokine storm” (Conti et al., 2020). Agents that target immune modulation rather than directly virucidal activity suggest that they may offer exciting targets for pharmacological intervention. Bromelain is a crude extract of the pineapple that is considered as a food supplement and is freely available to the general public in health food stores and pharmacies around the world (Pavan et al., 2012). Bromelain is also demonstrated to improve oral bioavailability of quercetin up to 80% similar to vitamin C (Manach et al., 2005; Harwood et al., 2007). Hence, a combination of quercetin, vitamin C and bromelain was preferred in this study, and none of the advers effects related to QCB supplement was observed in participants. SARS CoV2 can affect many other systems along with the lung, and its effect may last after the acute period of infection resolves. It can cause endothelial dysfunction, hypercoagulability, and cytokine storm (Potus et al., 2020). Quercetin mechanisms of action overlap with COVID-19 pathophysiology (Table 6) (Pearce et al., 1984; Bindoli et al., 1985; Hubbard et al., 2003; Pignatelli et al., 2005; Kumazawa et al., 2006; Shaik et al., 2006; Palmer et al., 2007; Boots et al., 2008; Loke et al., 2008a, 2008b; Romero et al., 2009).

**Table 6:** Quercetin’s mechanism of action.

Effect	Mechanism of action
Antioxidative capacity and blood vessel protection	Most potent scavenger of reactive oxygen species including superoxide, and reactive nitrogen species such as nitric oxide (Boots AW et al., 2008) Reduces the level of LDL oxidation, likely through inhibiting neutrophil myeloperoxidase (Loke WM et al., 2008a) Inhibition of the xanthine dehydrogenase/xanthine oxidase system is another important mechanism by which quercetin might decrease oxidative injury occurring after ischemia or other pathological conditions (Bindoli A et al., 1985) Improves endothelial function by inhibiting endothelin-1 effects including increased protein kinase C (PKC) activity induced by endothelin-1(Romero M et al., 2009)
Antiallergic and anti-inflammatory activities	Inhibits histamine release by affecting intracellular calcium levels and PKC activation (Pearce FL et al., 1984) Decrease in the release of tryptase, monocyte chemotactic protein-1 and IL-6 and the downregulation of histidine decarboxylase (Shaik YB et al., 2006) A potent inhibitor of leukotriene B4 formation in leukocytes (Loke WM et al., 2008b) Suppresses the production of TNF-alpha and nitric oxide by macrophages, microglial cells and mast cells stimulated with lipopolysaccharide (Kumazawa Y et al., 2006)
Vasodilatative effects	Phosphodiesterase-type 5-inhibitory effects (Palmer MJ et al., 2007)
Antiplatelet activity	Inhibits thrombin-induced and collagen-induced platelet activation (Hubbard GP et al., 2003) Down-regulation of CD40L on platelets and interference with adhesion molecules (Pignatelli P et al., 2005)

COVID 19 has an asymptomatic incubation period averaging 5–6 days (up to 14 days). Symptoms most frequently include fever, cough, anosmia, dysgeusia, and fatigue, with the possible onset of sputum production, headache, hemoptysis, diarrhea, dyspnea, and/or lymphopenia (Lei et al., 2020). In this study, most common symptoms at presentation were fatigue (62.5%), cough (60.4 %), anorexia (56.6%), thirst (54.3%), respiratory distress (51%), and chills (48.3%), respectively (Table 1). To our knowledge, thirst has not been reported as a symptom of COVID-19 in the literature so far.

COVID 19 is also associated with mental and neurological manifestations, including delirium or encephalopathy, agitation, stroke, meningo-encephalitis, anxiety, depression, and sleep problems. Most people with COVID 19 develop only mild (40%) or moderate (40%) disease; approximately 15% develop severe disease that requires oxygen support, and 5% have critical disease with complications such as respiratory failure, acute respiratory distress syndrome (ARDS), sepsis and septic shock, thromboembolism, and/or multiorgan failure, including acute kidney injury and cardiac injury. Computed tomography (CT) images of hospitalised patients with complications of COVID 19 reveal the presence of pneumonia with evidence of pulmonary ground-glass opacities, and severe cases may progress to ARDS and acute cardiac damage (Lei et al., 2020).

CT findings of the group receiving QCB treatment were statistically more severe than the group receiving standard therapy. Oxygen support was needed in 32.4% of the patients in the standard treatment group and 51% of the QCB treatment group. In the standard treatment group, 8/380 patients required intensive care due to respiratory failure. Although it was not statistically significant, the patient group who received QCB did not require intensive care due to respiratory failure (Table 5).

A significant and unique characteristic of the disease is venous thromboembolism (VTE), which manifests as a related coagulopathy that shows unique characteristics (Marchandot et al., 2020). Fauvel et al. (2020) reported on the largest cohort; 1240 patients in French hospitals and a confirmed incidence of 8.3% (PE confirmed by computed tomography pulmonary angiography). Early pathogenesis in COVID-19 pneumonia defined by a widespread endotheliilitis affecting multiple organ systems when viral inclusion bodies are observed within endothelial cells accompanied by apoptosis, inflammatory cell infiltration and microvascular thrombosis, well-described mechanisms associated with infection/inflammation, and, finally, the profound hypoxaemia that is often observed is a likely driver of vasoconstriction, inflammation and thrombosis. Although there was no significant difference between the two groups in terms of reduction in D-Dimer levels with treatment, in the standard treatment group, 3/380 patients required intensive care due to stroke, 2/380 patients due to myocardial infarction, and 1/380 patients due to delirium (Table 4, Figure 2). In the QCB treatment group, only 1/49 of the patients died in the ward as a result of myocardial infarction (Table 4, Figure 2). In the whole patient group, venous thromboembolism (VTE) rate was 1.4%. Therefore, we suggest that the combination of quercetin and standard anticoagulant therapy can also create a synergistic effect.

In a previous study, a rate of lymphopenia in 35%, thrombocytopenia in 12%, elevated CRP in 86%, increased LDH in 76%, elevated D-Dimer in 36% and increased ferritin in 63% were detected (Chen N et al., 2020). In our study, lymphopenia was detected in 20.7% of the cases, thrombocytopenia in 4.2%, high CRP in 32.6%, LDH in 54.3%, D-Dimer in 26.3% and Ferritin in 31.3% (Table 2).

In current study, although the lung involvement was more advanced and significantly comorbid COPD was present in the group with QCB supplement, a significant decrease was achieved in the acute phase reactants (APRs). The decrease in the levels of C-reactive protein and ferritin was significantly higher in the group that received standard treatment plus QCB compared to the other group (Table 4, Figure 2). Besides, QCB supplement is suggested to have a role on the elevation of the thrombocyte and lymphocyte count. The exaggerated release of the pro-inflammatory cytokines from ‘hyper-reactive’ monocytes thought to be the reason for the increase of APRs in COVID-19 (Askari et al., 2012). Therefore, those findings may be explained by the immune-modulatory properties of flavonoids on macrophages via contributing their transformation from pro- to antiinflammatory phenotypes (Conti et al., 2020). Monocytes play a critical role in the inflammatory response. Activated monocytes display relevant immunomodulatory activities, including the secretion of pivotal cytokines, such as pro-inflammatory cytokines interleukin (IL)-6, IL-1, IL-8, and tumor necrosis factor alpha (TNF-alpha). Different mechanisms may be involved in the abnormal activation of monocytes in chronic diseases (Kuznetsova T et al., 2020). Flavonoids have the ability to modulate macrophages from pro- to anti-inflammatory phenotypes, potentially contributing to the resolution of preestablished inflammatory processes (Mendes et al., 2019).

Variable bioavailability, high bio-transformations due to adsorption in the gut and complexity of the gut microbiota make it unlikely that flavonoids and their metabolites reach blood concentrations in the micromolar range (Pavan et al., 2012). We tried to overcome this problem with vitamin C and bromelain supplements. Although the pulmonary findings were more advanced in the patient group receiving QCB, it has a positive effect in terms of improvement in laboratory markers/results.

## 6. Conclusion

While there was no difference between the groups in terms of event frequency, QCB supplement group had more advanced pulmonary findings, and QCB supplement is shown to have a positive effect on laboratory recovery. We suggest that suboptimal bioavailability of QCB may explain this situation. Therefore, we conclude that further studies involving different doses, and plasma level measurements are required to reveal the dose/response relationship and bioavailability of QCB for a better understanding of the role of QCB in the treatment of SARS CoV-2.

## Sources of financial assistance

Financial support to provide the supplement that was used in this study was provided by the Laboratory of Orbitera Company. The study investigators had the primary responsibility for data collection, review, and management. The authors declare no associated financial or conflicting interests with the company.
